# EU Enlargement in Disregard of the Rule of Law: A Way Forward Following the Unsuccessful Dispute Settlement Between Croatia and Slovenia and the Name Change of Macedonia

**DOI:** 10.1007/s40803-022-00169-7

**Published:** 2022-03-28

**Authors:** Elena Basheska

**Affiliations:** CEU DI, Budapest, Hungary

**Keywords:** Rule of law, EU enlargement, Western Balkans, Bilateral disputes, Article 273 TFEU

## Abstract

EU enlargement has always been a political process. That said, the rule of law is an important aspect and principle of the EU enlargement policy. Implementation of EU driven reforms in candidate countries largely depends on the rule of law-based enlargement as well as on a clear EU perspective. Overpoliticisation of the enlargement process renders the EU’s enlargement law futile and undermines both the transformative effect of the pre-accession process and EU’s own values. The implementation of the enlargement condition for settlement of bilateral disputes, which became pronounced in the EU enlargement towards the Western Balkan countries, is having the negative effect of contributing to deterioration rather than promotion of the rule of law in both EU candidate countries and the EU’s enlargement process. Lack of predictability and rule of law accordingly, makes the effective application of the principle of conditionality impossible. A genuine reconsideration of the condition for settlement of bilateral disputes within the EU enlargement framework, clear EU perspective and viable way forward are urgently needed for bringing rule of law and the EU’s credibility on track.

## Introduction

According to Article 2 TEU, the rule of law is one of the fundamental values on which the EU is ‘founded’ and which is ‘common to the Member States’. In the words of the First Vice President of the Commission, Frans Timmermans, ‘[t]he rule of law is part of Europe’s DNA, it’s part of where we come from and where we need to go. It makes us what we are’.^1^

In its most recent Rule of Law Report, the European Commission stressed that the rule of law is ‘an important dimension and guiding principle for EU external action’^2^ and that ‘the EU will continue to pursue a strong and coherent approach in its external action, and in particular embed the rule of law in its work on enlargement, in the neighbourhood and globally’.^3^ Thus, the rule of law is equally important internally and externally—the main difference being in the available means of implementation: negative conditionality for internal usage as opposed to positive conditionality in EU external relations.^4^ In other words, while EU Member States are expected to respect the fundamental values to retain their membership rights in accordance with Article 7 TEU,^5^ candidate countries should respect such values to progress towards EU membership.^6^

Thus, Article 49(1) TEU, which is the main Treaty provision regulating EU enlargement, stipulates that ‘[a]ny European State which respects the values referred to in Article 2 [TEU] and is ‘committed to promoting them may apply to become a member of the Union’. Article 3(5) then stipulates that the Union shall ‘uphold and promote its values’ and ‘contribute to peace, security (…) as well as to the strict observance and the development of international law, including respect for the principles of the United Nations Charter’ in its relations with the wider world. Article 21(1) goes even further, emphasising that ‘[t]he Union’s action on the international scene shall be guided by the principles which have inspired its own creation, development and enlargement, and which it seeks to advance in the wider world’ including the rule of law principle and respect for the principles of the United Nations Charter and international law.

EU values, including essentially the rule of law principle are, therefore, combined with the principles of the UN Charter in the EU’s external action.^7^ The most fundamental principle of the UN Charter, if not *jus cogens*,^8^ is sovereign equality of states which enables states to enjoy their sovereign rights freely and imposes a duty on them to take into consideration the sovereign rights of other states.^9^ Disrespect of the fundamental principle of sovereign equality of states in the EU enlargement process and particularly with regard to settlement of bilateral disputes, which has become an important accession condition outside the Copenhagen criteria, would almost naturally undermine EU values and especially the rule of law principle. This further paves the way for overpoliticization of the enlargement process, rendering the EU’s enlargement law futile and undermining both the transformative effect of the pre-accession process and EU’s own values. The focus of candidate countries in such circumstances is not so much on the implementation of the necessary reforms but on overcoming political obstacles and hurdles to secure the support of EU Member States or please the Union—an aspect which has been often overlooked by EU institutions.^10^

Indeed, conditionality tied to financial assistance in the enlargement process has proved insufficient to achieve any substantial changes in priority sectors, such as the rule of law.^11^ Thus, despite many years of EU financial support to reforms in the Western Balkans, in 2018 the European Commission acknowledged the grave deficiencies in guaranteeing the rule of law in the region, stating that ‘countries show clear elements of state capture, including links with organised crime and corruption at all levels of government and administration, as well as a strong entanglement of public and private interests [which] feeds a sentiment of impunity and inequality’.^12^ It further noted that there is ‘extensive political interference in and control of the media [and that a] visibly empowered and independent judiciary and accountable governments and administrations are essential for bringing about the lasting societal change that is needed’.^13^ Four years later, the European Court of Auditors found that ‘while EU action has contributed to reforms in technical and operational areas, such as improving the efficiency of the judiciary and the development of relevant legislation, it has had little overall impact on fundamental rule of law reforms in the region’.^14^ The main reason for that, according to the European Court of Auditors, lies in the ‘insufficient domestic political will to drive the necessary reforms’.^15^ Overpoliticisation of the enlargement process and lack of predictability, however, have rarely been brought into direct connection with the weak reforms and absence of the rule of law in the Western Balkan countries. While the European Commission made some attempts^16^ to promote the enhancement of the accession process and offer a credible EU perspective for the Western Balkans, results are yet to be seen.

This article analyses the rule of law principle through the prism of predictability and critically assesses implementation of the enlargement condition for early settlement of bilateral disputes of candidate countries. Thus, Sect. 2 discusses overpoliticisation of the EU enlargement process and lack of predictability, focusing primarily on conditionality and absence of clarity with regard to the requirement for early settlement of bilateral disputes. Lack of clear EU perspective and legal obscurity of the condition for settlement of bilateral disputes in the enlargement process make the application of the principle of conditionality unworkable. As elaborated in Sect. 3, this drawback of the enlargement process has been also recognised by the European Commission which, however, has not been successful in implementing any meaningful changes to date. Indeed, the current situation^17^ and stories from the past are not particularly encouraging. Sections 4 and 5 analyse the pre-accession trajectories of two countries to tell the story of overpoliticisation of the enlargement process at the sacrifice of law: the stories of Croatia and (now) North Macedonia^18^ respectively—the first one being the newest EU Member State and the latter being a candidate country stuck in the EU’s waiting room for years. What’s more, stories of Croatia and North Macedonia are not exceptions but new reality of the enlargement process towards the Western Balkans which may be reasonably expected by other countries in the region given their troubled past and remaining disputes with EU Member States.^19^ Section 6, therefore, discusses possible international and EU mechanisms for settlement of bilateral disputes. It is argued that the EU enlargement framework does not offer an appropriate platform for settlement of bilateral disputes that fall outside the EU law. Instead, solution should be sought in making use of Article 273 TFEU which would allow states to settle their bilateral disputes on equal footing by employing CJEU, and enjoy better security with regard to the implementation and compliance with the final decision of that Court. This would help both the Union and candidate countries in overcoming current and future stalemates of the enlargement process owed to unresolved bilateral disputes. Drawing on previous sections, Sect. 7 presents the general conclusions of this article.

## Lack of Predictability in the EU Enlargement Process

The EU enlargement to the Western Balkans is a perfect example of the imperfect and highly politicised enlargement process. While Croatia joined EU after overcoming political struggles with neighbouring Slovenia and enjoying support from other Member States, other Western Balkan countries remain outsiders with blurry vision of the future. Indeed, the future EU integration of the Western Balkans is almost as uncertain now as over 18 years ago, i.e. before the Thessaloniki Agenda from 2003,^20^ which confirmed the EU accession perspective for the countries of the region. Almost two decades after committing to support the EU integration of the Western Balkan countries and welcome them in the future, EU Member States reaffirmed their support for the region at the EU-Western Balkans summit of 6 October 2021 with lots of hesitations.^21^ The attempt of the host, Slovenia, to convince EU Member States commit to a timeline for the future accession of the Western Balkan countries was, therefore, expectedly unsuccessful.^22^

The EU enlargement process has, therefore, become more unpredictable than ever before, depending more on domestic policies and EU circumstances than on the actual reforms of candidate countries in line with the established enlargement criteria. Yet, predictability is the key procedural rule of law component of the enlargement regulation.^23^ As such, it is inevitably connected to pre-accession conditionality, which in absence of predictability, could be hardly attainable. As more recently clarified by the European Commission, predictability, and conditionality in the enlargement process mean ‘ensur[ing] greater clarity on what the Union expects of enlargement countries at different stages of the process, and what the positive and negative consequences are of progress or lack thereof’.^24^ As further confirmed by the Commission, ‘[t]he core element of the merit-based accession process is its conditionality. However, in order to achieve that, conditions must be clear from the outset. (…) These conditions must be objective, precise, detailed, strict and verifiable’.^25^

That said, the condition for settlement of bilateral disputes has neither been clear from the outset, nor is it ‘objective, precise, detailed, strict and verifiable’,^26^ which makes the pre-accession process highly unpredictable as no assessment criteria for fulfilment of the condition for settlement of disputes exist. In other words, progress of countries towards membership depends on satisfaction of involved Member States from the outcome of the dispute settlement rather than on any ‘objective, precise, detailed, strict and verifiable’ condition. The opportunities for Member States to exert pressure in the enlargement process can vary ‘from withholding consent to the opening of negotiating chapters, to objecting to the graduation of a country to a new phase (candidate membership, opening of negotiations, membership)’.^27^ Indeed, Member States have a crucial role in the enlargement process. As put by Kochenov, ‘[i]t is notable that the [EU] Institutions, although taking part in the process of enlargement preparation, do not sign the Treaties of Accession. Clearly, enlargement of the EU is not about the Union enlarging but about the Member States enlarging the Union with the help of Institutions’.^28^ Yet, allowing Member States to redefine enlargement criteria in line with their domestic preferences and politics does not only undermine the rule of law as a fundamental part of the enlargement regulation, but also discredits the entire enlargement process.

Such anomalies of the enlargement process have been best revealed through the bilateral disputes involving Western Balkan countries, even if the new requirement for settlement of bilateral disputes started to crystallise in the EU enlargement policy after the establishment of the Copenhagen criteria in the view of the fifth enlargement round.^29^ The new requirement was introduced in response to EU security considerations with respect to the unresolved issues of the applicant countries, which included border issues and questions related to the protection of minorities.^30^ The EU’s approach largely corresponded with peaceful settlement of disputes and other principles of international law. Those disputes, however, involved mainly candidate countries.

The balanced and consistent EU approach started changing with the Western Balkans moving towards membership. Unlike bilateral disputes in the fifth enlargement round, bilateral disputes of Western Balkan countries involved not only candidate countries but EU Member States too. Yet, once EU Member States are involved, things naturally become more complicated as their national interests are affected. In most cases, bilateral disputes involve sensitive matters for which there is a general consensus in national parliaments. Politicians build their careers around such disputes and negative outcome would most certainly mean political suicide. Thus, for EU Member States, resolving bilateral disputes within a proper legal framework between equal states may be less attractive than achieving favourable outcome by using membership powers within a framework where they can largely shape the rules.

Initially distancing itself from bilateral disputes between neighbouring countries and considering it ‘inappropriate’^31^ to make membership dependent on the settlement of such disputes, even the European Commission bowed to the pressure of EU Member States.^32^ Indeed, despite recognising that ‘[b]lockages linked to bilateral issues can compromise the transformative power of the enlargement process,’^33^ the Commission required early solutions to the bilateral issues during the enlargement process over a decade ago.^34^ It then, however, struggled to find a balance between the rule of law in the enlargement process and ambitious interests of Member States in the context of the EU enlargement towards the Western Balkan countries, sending an unclear message as to whether settlement of bilateral disputes that fall essentially outside the scope of EU law is an enlargement criterion, and if it is, what specifically is expected from candidate countries.

This makes the requirement for settlement of bilateral disputes vague and confusing rather than ‘objective, precise, detailed, strict and verifiable’ which does not only call into question the credibility of the EU accession process based on ‘strict but fair conditionality and the principle of own merits’^35^ but also puts at risk sustainability of the reform efforts of candidate countries.^36^

## Attempt for Improvement

In its 2020 Communication aimed at ‘reinvigorating the accession process’,^37^ the European Commission sounded almost romantic, noting that ‘[t]he European Union and its Member States have consistently, since the Feira and Thessaloniki Summits in 2000 and 2003, expressed their unequivocal support for the European perspective of the Western Balkans’.^38^ It further noted that ‘[t]he Council conclusions adopted at the General Affairs Council in June 2019 has also reaffirmed *“its commitment to enlargement, which remains a key policy of the European Union* (…)*”*’.^39^

The Commission then emphasized that ‘[t]his firm, merit-based prospect of full EU membership for the Western Balkans is in the Union’s very own political, security and economic interest’.^40^ Therefore,

‘[a] credible accession perspective is the key incentive and driver of transformation in the region and thus enhances our collective security and prosperity. It is a key tool to promote democracy, rule of law and the respect for fundamental rights, which are also the main engines of economic integration and the essential anchor for fostering regional reconciliation and stability. Maintaining and enhancing this policy is thus indispensable for the EU’s credibility, for the EU’ success and for the EU’s influence in the region and beyond—especially at times of heightened geopolitical competition’.^41^

Weaknesses of the enlargement process were also emphasised by the Commission. In particular, it has been noted that:

‘[it] is of major importance to build more trust among all stakeholders and to enhance the accession process and make it more effective. It has to become more predictable, more credible**—**based on objective criteria and rigorous positive and negative conditionality, and reversibility—more dynamic and subject to stronger political steering.^42^

Indeed, predictability is the core rule of law component of the enlargement regulation, in the absence of which successful implementation of pre-accession criteria through conditionality is hardly possible. The perspective of EU accession is the most important incentive for aspiring countries and, as such, is of significant importance for the implementation of the necessary reforms, including the rule of law related reforms, in these countries.

For the purpose of strengthening the entire accession process, the EC Communication set out the Commission’s concrete proposals. The core objective of the EU with regard to the Western Balkans countries was said to be their preparing to meet the membership criteria including ‘supporting fundamental democratic, rule of law and economic reforms and alignment with core European values’^43^ which will ‘in turn foster solid and accelerated economic growth and social convergence’.^44^ The accession process was to be reinvigorated through: (a) more credibility; (b) a stronger political steer; (c) a more dynamic process; (d) predictability, positive and negative conditionality.

More credibility meant that the accession process ‘needs to rest on solid trust, mutual confidence and clear commitments on both sides’. It meant that ‘the Western Balkans leaders must deliver more credibly on their commitment to implement the fundamental reforms required’.^45^ It also meant that:

‘the European Union delivers on its unwavering commitment to a merit-based process. When partner countries meet the objective criteria and the established objective conditions, the Member States shall agree to move forward to the next stage of the process. All parties must abstain from misusing outstanding issues in the EU accession process’.^46^

Credibility was to be reinforced through:

‘even stronger focus on the fundamental reforms (…) [n]egotiations on the fundamentals will be opened first and closed last and progress on these will determine the overall pace of negotiations. Negotiations on the fundamentals will be guided by:

- roadmap for the rule of law (…).
- [a] roadmap on the functioning of democratic institutions and public administration reform.
- [a] stronger link with the economic reform programme process to help the countries meet the economic criteria’.^47^

A stronger political steer meant that countries aspiring to join EU ‘must reflect an active societal choice on their part to reach and respect the highest European standards and values’.^48^ It also meant that ‘both sides should show more leadership and live up to their respective commitments in public, while coming in more directly on matters of concern’.^49^

A more dynamic process meant organisation of chapters in thematic clusters which will cover broad themes and ‘will allow a stronger focus on core sectors in the political dialogue and provide an improved framing for higher level political engagement’.^50^ This was intended to allow for the ‘most important and urgent reforms per sector to be identified’,^51^ giving the reform processes new impetus.

For the purposes of predictability, the Commission ‘will use the enlargement package to check the compliance of the candidates with the acquis and provide clearer guidance on specific reform priorities and alignment criteria as well as expectations for next steps in the process’.^52^ Clarity of accession conditions has been said to be of particular importance to achieve conditionality in the merit-based accession process. In particular:

‘[i]t is important that candidate countries know the benchmarks against which their performance will be measured and that Member States share a clear understanding of what exactly is requested from the candidates. The Commission will better define the conditions set for candidates to progress, in particular through its annual reports. These conditions must be objective, precise, detailed, strict and verifiable. The Commission will also use third party indicators where relevant to provide Member States with the broadest possible base for their decisions’.^53^

Intertwinement between the key component of the enlargement regulation, predictability, and successfulness of conditionality in the pre-accession process has been thus well recognised by the European Commission. The plan was clear: good progress of Western Balkan countries in implementing reforms would lead to closer integration with the EU, increased funding and investments, while backsliding could lead to sanctioning. Yet, not much has been done for the promotion of the rule of law in the light of the condition for settlement of bilateral disputes in the pre-accession process since the EC 2020 Communication. Indeed, unpredictability has become the most predictable aspect of the EU enlargement policy, despite the efforts of the European Commission to reinvigorate the process. This is notwithstanding the fact that the condition for settlement of bilateral disputes has shown to be of greater significance than any other well established enlargement criterion making progress of candidate countries dependent on satisfactory solutions – be that before or during the opening of the accession negotiations.^54^

Shortly after the EC 2020 Communication, in March 2020, the European Commission welcomed ‘the Council’s decision to open accession negotiations with Albania and North Macedonia, subject to final endorsement by the European Council members’.^55^ The President of the European Commission, Ursula von der Leyen, was quick with praising the EU achievement, stating that:

‘The European Union delivers on its promise. North Macedonia and Albania did what was asked of them and they have continued making progress in the reforms needed. Today marks the start of the journey to a bigger and stronger European Union. And this decision is in the European Union’s geostrategic interest’.^56^

The Commissioner for Neighbourhood and Enlargement, Olivér Várhelyi, was also loud on the subject matter:

‘Opening of accession talks sends a loud and clear message not only to the two countries, but to the Western Balkans as a whole. It reaffirms and delivers on the EU’s commitment to the European perspective of the region: its present is with the EU and its future is in the EU. The Commission will move quickly and propose soon the draft negotiating frameworks with the two countries integrating the elements of the revised methodology. Today’s decision confirms the geostrategic importance of the Western Balkans and demonstrates that Europe is willing and able to take geopolitical decisions even in these trying times of corona virus pandemic’.^57^

Almost 2 years after the EC 2020 Communication and the ecstatic statements of the EU officials, however, neither Albania, nor North Macedonia have started their EU accession negotiations.^58^ While the problem of bilateral disputes has been addressed on numerous occasions, and most recently in the 2021 Communication on EU Enlargement Policy,^59^ the enlargement criterion for settlement of such disputes still begs for detailed clarification. Statements that settlement of disputes is ‘crucial’^60^ and that bilateral disputes need to be resolved ‘as a matter of priority,’^61^ or that existing bilateral agreements should be ‘implemented in good faith’^62^ and that ‘differences must be resolved through peaceful dialogue and in accordance with international law’^63^ are vague and unhelpful, to which the two disputes discussed in the following sections testify.

## Problem with the Next Door Neighbour: The Border Dispute Between Croatia and Slovenia

The progress of the Western Balkan countries towards EU accession has been largely determined by their relations with EU Member States and particularly with neighbouring states to which the border dispute between Croatia and Slovenia testifies. While not being unique to halting the enlargement process,^64^ the border dispute between Croatia and Slovenia is the first instance where the EU formally brokered between two parties on the pretext of unblocking the EU enlargement process.^65^

### Attempt for Settlement of the Border Dispute Before an Arbitral Tribunal

The border dispute between Croatia and Slovenia relates to a boundary demarcation of a small gulf in the northeast extreme of the Adriatic Sea. The bilateral dispute between the two countries, which caused a delay to Croatia’s accession negotiations, was not made an issue at the time of the Slovenian accession. As previously noted, the EU did not apply conditionality strictly to the settlement of bilateral disputes in the fifth enlargement. It did become an important issue for Slovenia, however, once that country joined the Union and left Croatia behind.^66^

The Negotiating Framework for Croatia provided explicitly that the advancement of the negotiations with that country will be guided by its progress in preparing for accession, measured in particular against ‘Croatia’s commitment to good neighbourly relations’^67^ and its ‘undertaking to resolve any border disputes in conformity with the principle of peaceful settlement of disputes in accordance with the United Nations Charter, including if necessary compulsory jurisdiction of the International Court of Justice’.^68^ As rightly argued by Avbelj and Letnar Černič, the delimitation dispute between the two neighbouring countries involved crucially a sensitive political question. However as further discussed by the authors:

‘political questions cannot be solved within a vacuum allowing arbitrary and one-sided measures based on the maxim of the rule of the most powerful. Political questions have to be resolved within the realm of law. The role of the law—and by speaking about sovereign states we are primarily and foremost in the field of international law—is to enable, to facilitate, and to safeguard a peaceful political discourse the result of which, if it remains within the valid framework of the legal discourse, is a legally valid and acknowledged outcome’.^69^

Although both Slovenia and Croatia wanted to see a settlement of the bilateral dispute, they disagreed on the method which was to be used to reach a solution. Assuming to have the law on its side, Croatia was in favour of using the ICJ for the settlement of the dispute, while Slovenia preferred a ‘[political] mediation instead of judicial arbitration’.^70^ Stepping out of the legal framework, Slovenia employed its EU membership powers to achieve political gains by securing non-juridical arbitration, which promised the country a more favourable outcome. To secure its position, Slovenia effectively blocked the opening or closing of fourteen negotiation chapters with Croatia, not all of them connected to compliance with the accession criteria.^71^

Ultimately, on 4 November 2009, the two parties signed an Arbitration Agreement under the EU auspices including specific provision related to the pre-accession process.^72^ Thus, Article 9 of the Arbitration Agreement stipulated that ‘Slovenia shall lift its reservations as regards opening and closing of negotiation chapters where the obstacle is related to the dispute’^73^ and that ‘[b]oth Parties shall refrain from any action or statement which might negatively affect the accession negotiations’.^74^ On 1 July 2013,^75^ Croatia became the newest Member State of the EU.^76^ The Commission applauded the launch of the arbitration process between Slovenia and Croatia despite the fact that the Arbitration Agreement meant defeat for the rule of law in the enlargement process. In the words of the (then) European Commissioner for Enlargement, Štefan Füle, the ‘common agreement [was] a very welcome signal for the positive development of the good neighbourly relations between the two countries as well as for the Western Balkans regions showing that even difficult issues can be best solved by means of dialogue and cooperation’.^77^

The Arbitration Agreement, however, was rather a result of the pressure on the candidate country in the pre-accession process and imposition of the Member State involved in the bilateral dispute than of good neighbourly dialogue and cooperation between the two neighbouring countries. Yet, as already noted, settlement of disputes under pressure and outside the proper legal framework may not be sustainable in the long run. This is particularly the case when circumstances or position of involved countries change to which further developments in the arbitration process between Croatia and Slovenia testify. Specifically, the settlement of the bilateral dispute between Croatia and Slovenia before the Arbitral Tribunal did not go as planned. The award of the Arbitral Tribunal which should have been binding on the two parties and should have constituted a definitive settlement of the dispute,^78^ has not come into force. This was substantially due to a violation of the Arbitration Agreement by Slovenia but also due to the fact that Croatia was already an EU Member State at that time, free of threats for blockages of its EU membership.

Article 4 of the Arbitration Agreement determined the applicable law to include: (a) the rules and principles of international law; (b) international law, equity and the principle of good neighbourly relations in order to achieve a fair and just result by taking into account all relevant circumstances for determining the maritime and land boundary between the two countries. Article 6(2) of the same Agreement envisaged that the proceedings shall be conducted in accordance with the Permanent Court of Arbitration Optional Rules for Arbitrating Disputes between Two States. According to these rules, appointed arbiters shall be independent and impartial.^79^ Article 10(1) of the Arbitration Agreement obliged both parties to ‘refrain from any action or statement which might intensify the dispute or jeopardize the work of the Arbitral Tribunal’.^80^ The Arbitral Tribunal could order any provisional measures it deemed necessary to preserve the stand-still if it considered that circumstances so required.^81^ Finally, and of particular importance for the further developments in the case, Article 6(5) of Agreement stipulated that ‘the proceedings are confidential’.

While both states seemed determined to resolve their dispute when signing the Arbitration Agreement, things started to go wrong after two statements of the Minister of Foreign Affairs, Karel Erjavec, which triggered the alarm for Croatia. On 7 January 2015, Erjavec publically stated that he ‘had talks in The Hague last year … And [he] made it very clear to the Arbitral Tribunal that if they do not fulfil this task—[they] in Slovenia shall consider that the Arbitral Tribunal has not executed its mandate. Because the contact with the high seas has not been determined’.^82^ On 22 April 2015, Erjavec publically stated the following:

‘According to the information that I have, which is very much unofficial, as well as on the basis of a feeling that our legal team has, being composed of the world’s best renowned scholars of the law of the sea, we are somehow optimistic in a way that the Arbitral Tribunal will determine that contact with the high seas’.^83^

These statements were enough for Croatia to take action almost immediately after the second statement of Mr Erjavec. On 30 April 2015, the then First Deputy Prime Minister and Minister of Foreign and European Affairs of Croatia, Vesna Pusić, sent a letter to the Arbitral Tribunal expressing deep concern over the two statements of the Slovenian Minister of Foreign Affairs and of the previous conclusion of the Slovenian Assembly:

‘Croatia is deeply troubled by language of both statements, which could be construed as implying that one of the Parties to the proceedings may have an informal channel of communication with the Tribunal that may compromise the arbitration procedure and its outcome.

Moreover, taken together, these statements could also be seen as being intended to seek to bring pressure on the Tribunal. This has been a tendency also in the earlier phase of the proceedings when, on 4 February 2013 the Slovenian Parliament adopted the Conclusion according to which: “The Republic of Slovenia declares that the task of the Arbitration Tribunal is to determine territorial contact of the territorial sea of the Republic of Slovenia with the High Seas (contact of Slovenia to the High Seas)”, and also stated that Slovenia will consider any decision of the Arbitration Tribunal that would not ensure this as “a decision ultra vires (in contravention of the mandate of the Arbitration Tribunal)”’.^84^

Subsequently, the Tribunal expressed its concerns in its response to the letter of Croatia, recalling that ‘[t]he Parties shall not engage in any oral or written communication with any member of the Arbitral Tribunal ex parte in connection with the subject matter of the arbitration or any procedural issues that are related to the proceedings’.^85^ However, the Tribunal concluded that ‘no information about the likely outcome of any aspect of the arbitration has been disclosed’.^86^

On the 22 July 2015, leaked audio recordings and excerpts of conversations between a Member of the Arbitral Tribunal appointed by Slovenia and the Agent of Slovenia before the Arbitral Tribunal were published first in Serbian and then in Croatian media outlets,^87^ revealing, ‘strategies to influence other members of the Tribunal and to manipulate with the file of the case’ as described by Croatia.^88^

Although both the Member of the Arbitral Tribunal appointed by Slovenia and the Agent of Slovenia before the Arbitral Tribunal resigned from their positions immediately,^89^ Croatia considered that the two resignations did not sufficiently address the issue and that ‘the entire arbitral process has been tainted’.^90^ After clarifying its position about the circumstances, Croatia asked that the Tribunal suspends the proceedings immediately and shortly after that, the Croatian Parliament adopted a decision which has obliged the Croatian Government to start the termination of the Arbitration Agreement with Slovenia.^91^ The Government of Croatia proceeded with the termination of the Agreement which marked the beginning of a new struggle between the two neighbouring countries.^92^

The European Commission also voiced its opinion on the issue, emphasising that ‘setting of borders between Member States does not fall within the competence of the Union’^93^ but also noting that border disputes ‘can have an impact on the application of EU law’^94^ and should be, therefore, resolved quickly. The continuation of the process in front of the Tribunal, however, has been rejected by Croatia ever since the termination of the Agreement.^95^ The country proposed instead ‘to proceed without delay in identifying an alternative way to resolve the territorial dispute’, which has been in turn rejected by Slovenia.^96^

Continuing its work on the matter, the Tribunal confirmed the interconnection between the enlargement process and the settlement of the dispute in its Partial Award:

‘At the time, negotiations were developing with respect to the accession of Croatia to the European Union. Slovenia had expressed reservations as regards the opening and closing of some of the negotiation chapters. It accepted, in Article 9 of the Arbitration Agreement, to lift those reservations. Indeed, the Agreement is intimately tied to the process of Croatia’s accession to the European Union; (…) The Agreement was negotiated with the full support of the European Union, and the Presidency of the Council of the European Union witnessed the signature of the Agreement. Thus, a nexus was established between the settlement of the territorial and maritime dispute and the accession of Croatia to the European Union’.^97^

Furthermore, the Tribunal found that Slovenia has violated the Arbitration Agreement,^98^ but concluded that ‘the breaches of the Arbitration Agreement by Slovenia do not render the continuation of the proceedings impossible and, therefore, do not defeat the object and purpose of the Agreement’.^99^ It has been, therefore, concluded by the Tribunal that Croatia could not terminate the Agreement under Article 60 (1) of the Vienna Convention and that the Arbitration Agreement remained in force.^100^ Croatia, however, has seen the ‘Arbitral Tribunal’s Partial Award as a missed opportunity for the Arbitral Tribunal to restore confidence in independence and impartiality of its own work, as well as confidence in international arbitration as such’,^101^ emphasising also that:

‘Croatia is no longer a party to the arbitration process, so, accordingly, the Ministry of Foreign and European Affairs shall not comment on the intentions or decisions of the Arbitral Tribunal, nor does Croatia consider itself bound by them, no matter if they concern procedural matters or the merits of the border dispute between the two states’.^102^

While Croatia explicitly rejected to accept any further involvement in the process, the Tribunal continued with its work on the case and announced the Final Award on 29 June 2017.^103^ The decision of the Tribunal was expressly rejected by Croatia^104^ and the Final Award could not be implemented without the participation of both sides.^105^

### Attempt for Settlement of the Dispute Before the CJEU

The end of the arbitration process marked the beginnings of a power struggle between the then two EU Member States. Thus, on 8 September 2017, Slovenia objected to Croatia’s bid for OECD membership^106^ and one week later, it initiated legal action against the European Commission for granting Croatia a derogation enabling it to use ‘Teran’ on Croatian wine labels under certain conditions, despite the fact that Slovenia had Protected Designation of Origin for ‘Teran’.^107^ The rues of play were different this time despite the attempts of Slovenia to use the same power tactics as in the pre-accession process. Croatia has become an EU Member States well before the termination of the Agreement in 2015, gaining same position and membership powers as neighbouring Slovenia and thus being able to abandon the arbitration process without blockages or other severe consequences.

After a number of unsuccessful attempts to agree with Croatia and numerous maritime incidents, Slovenia brought the matter before the Commission, initiating a procedure for a declaration of failure to fulfil obligations against Croatia in accordance with Article 259(2) TFEU. Wanting to stay ‘neutral’^108^ or rather not wanting to deal with the sensitive issue, the European Commission has chosen not to issue reasoned opinion in the following three months as provided by Article 259(4) TFEU and contrary to the legal opinion of the Commission’s Legal Service on the border dispute between Croatia and Slovenia which has been kept out of the public eye.^109^

‘Der Spiegel’,^110^ however, published the legal opinion of the Commission’s Legal Service, revealing its contents. The Commission’s Legal Service emphasised in its legal opinion that it ‘considers that most of the heads of claim put forward by Slovenia in order to establish a breach of EU law by Croatia are established’^111^ and that ‘the Commission must observe that there is an arbitration agreement … Therefore, the outcome of the arbitration procedure must be respected by the EU, and provisions of EU law must be interpreted in the light of it’.^112^ The legal opinion of the Commission’s Legal Service was, however, put aside. This move of the Commission has been largely criticised and seen as politically motivated. In the words of Irena Joveva, a Slovenian MEP, ‘[i]t appear[ed] that the Juncker Commission consciously ignored the opinion of its Legal Service, and serious questions have arisen as to whether its actions were politically motivated’.^113^

On the 13 July 2018, Slovenia brought direct action against Croatia claiming a number of violations of EU law resulting from the failure of Croatia to recognise the final award of the Tribunal. In particular, Slovenia requested the CJEU to declare that Croatia has failed to fulfil *inter alia* its obligations under:

1. the principle of sincere cooperation enshrined in Article 4(3) TEU, ‘in that it has jeopardised the attainment of the objectives of the European Union, in particular peace building and ever closer union among the peoples of Europe, and has prevented the Republic of Slovenia from complying with its obligation to implement EU law fully throughout its territory’;
2. ‘the principle of the rule of law, enshrined in Article 2 TEU, which is an essential condition of membership of the European Union and obliges the Republic of Croatia to respect the territory of the Republic of Slovenia as determined by the final award made on 29 June 2017 by the tribunal established in the arbitration procedure relating to the territorial and maritime dispute between those two States (…), in accordance with international law’.^114^

In addition, Slovenia has argued that Croatia infringed: Regulation (EU) No 1380/2013 on the Common Fisheries Policy by refusing to implement the reciprocal access regime and preventing Slovenians from enjoying rights as provided for by that regulation; the system of control, of inspection and of implementation of the rules as provided for by Council Regulation (EC) No 1224/2009 and Implementing Regulation (EU) No 404/2011 of 8 April 2011 by preventing Slovenia to carry out its tasks under the system and not respecting jurisdiction rights of Slovenia; Regulation (EU) 2016/399 on a Union Code on the rules governing the movement of persons across borders; and Directive 2014/89/EU establishing a framework for maritime spatial planning by adopting and implementing the ‘Spatial planning strategy of the Republic of Croatia’.^115^

Croatia asked the CJEU to dismiss Slovenia’s action as inadmissible on the basis that alleged infringements were ‘ancillary to settlement of the dispute concerning the validity and legal effects of the arbitration agreement and the arbitration award’^116^ and accordingly, ‘the Court lacks jurisdiction to rule on the infringement of obligations arising from EU law if those obligations are ancillary to prior settlement of another dispute that does not fall within the jurisdiction of the Court’.^117^ As argued by Croatia, the real subject matter of the dispute related to the validity and legal effects of the arbitration agreement which falls outside EU law, and of the arbitration award which has not been implemented.^118^ Croatia thus argued that the Court lacked jurisdiction ‘under Article 259 TFEU to rule on the validity and effects of either the arbitration agreement, which is an international agreement not forming an integral part of EU law, or the arbitration award made on the basis of that agreement’.^119^

The CJEU agreed with Croatia, concluding that ‘the infringements of EU law pleaded are ancillary to the alleged failure by the Republic of Croatia to comply with the obligations arising from a bilateral international agreement to which the European Union is not a party and whose subject matter falls outside the areas of EU competence’.^120^ As a consequence, the CJEU confirmed that it lacks jurisdiction to rule in the action brought by Slovenia.^121^ The CJEU thus set the infringement threshold triggering action either under Article 258 TFEU or 259 TFEU above ‘ancillary’ connections between the alleged breach of EU law and obligations under the bilateral treaty that causes a breach.^122^ The fact that the EU witnessed the Arbitration Agreement and participated in its conclusion was not ‘sufficient for the arbitration agreement and the arbitration award to be considered an integral part of EU law’.^123^

In other words, ‘subject matter of an action for failure to fulfil obligations brought under Article 259 TFEU can only be non-compliance with obligations arising from EU law’,^124^ rather than non-compliance with obligations falling outside the scope of EU law. This is even if anticipated compliance by the candidate country with such obligations has been made important EU accession criterion and the relevant EU Accession Treaty has been modified in line with such anticipation.^125^ Put differently, settlement of disputes that fall outside the scope of EU law may not be sustainable in the long run unless there is a strong determination, will and support on both sides for such settlement and the methods used. Once the candidate country becomes an EU Member State, enforcement of the bilateral treaty or award cannot be guaranteed given the problems of enforceability of international decisions and lack of jurisdiction of the CJEU.^126^

Furthermore, in its previous case law, the CJEU held that ‘it lacks jurisdiction to give a ruling on (…) interpretation (of international Convention), and on the obligations arising under it for the Member States since (…) it was an international agreement concluded by the Member States which did not form an integral part of Community law’.^127^ In *Slovenia* v *Croatia*, however, the Court made a step further by effectively setting aside the Final Award of the arbitral tribunal by encouraging the two Member States ‘to strive sincerely to bring about a definitive legal solution consistent with international law (…) that ensures the effective and unhindered application of EU law in the areas concerned, and to bring their dispute to an end by using one or other means of settling it’.^128^ Yet, it should be recalled that the Arbitral Tribunal already issued its Final Award and determined the borders between the countries, deciding also previously that ‘Croatia was not entitled to terminate the Agreement’^129^ and that the ‘Arbitration Agreement remains in force’.^130^ To put it another way, the mechanism for settlement of the bilateral dispute supported, if not imposed upon the candidate country in the pre-accession process, as well as the outcome of the entire process have been largely discarded post-accession.

Last but not least, to agree with McGarry, by deciding that it lacks jurisdiction in *Slovenia* v *Croatia*, the CJEU mapped out the rule of law principle and the principle of sincere cooperation as general principles which may be invoked in infringement procedures only when there is first a violation of a more specific obligation under EU law.^131^ In particular, Slovenia alleged that Croatia infringed Article 2 TEU, rejecting to abide by the rule of law by failing to comply with the arbitration award contrary to the commitment entered into during the EU accession process and that it has infringed, in that respect, the principles of sincere cooperation and *res judicata*. Rather than looking into *pacta sunt servanda* as a general principle of law and a component of the rule of law principle,^132^ the CJEU went on to conclude that in order to verify the alleged violation of the rule of law principle, it would have had to examine the extent and limits of the territories of Croatia and Slovenia in the light of the arbitration award which went beyond its powers.^133^ While supremacy of EU law and non-applicability of the *pacta sunt servanda* principle to bilateral treaties between Member States that are in conflict with EU law have been long established,^134^ the case of *Slovenia v Croatia* was rather different concerning alleged infringement of a bilateral treaty that formed an essential part of the accession negotiations rather than involving conflict of EU law. Notwithstanding the reasons of the Court—be that the political sensitiveness of the issue or politicisation of the case,^135^ the contaminated arbitration process or the unfair treatment of Croatia in the pre-accession process—the CJEU ruling has paved the way for future instances of non-compliance with obligations arising from bilateral treaties, international decisions and arbitration awards alike.^136^

## When the Rule of Politics Replaces the Rule of Law: The Name Dispute between Greece and Macedonia

The Macedonian name saga has lasted for almost three decades before being recently resolved under significant international pressure, if not direct EU intervention. The story of the settlement of the name dispute is not one that EU should be particularly proud of. It is a story about the triumph of politics over the rule of law in the enlargement process and devaluation of EU values. Yet, the final solution to the dispute may be unsustainable in the long run considering the issues that may arise from disrespect for the law and potential change of circumstances in the future.

Macedonia successfully escaped the armed conflicts that followed the break-up of Yugoslavia. However, an unsolved dispute between Greece and Macedonia over the name of the latter became problematic as the country started to move slowly towards NATO and EU. Since the early 1990s, the name of the newly independent state became one of the dominant issues in the politics of the neighbouring countries both of which involved in lengthy negotiations.^137^ The dispute over the name occurred at a variety of levels and in a variety of contexts.^138^ The newly independent country initially faced problems with its admission to the UN and with its recognition by EU Member States which led to the saga with its prospective accession to NATO and the EU.^139^ Following the admission of Macedonia to the UN, that organisation undertook bringing the two parties to an agreement through special mediators. In 1995 an Interim Accord was signed which regulated the conduct of the two countries and became the main framework for development of their relations.

The two countries ‘agree[d] to continue negotiations under the auspices of the Secretary-General of the United Nations pursuant to Security Council resolution 845 (1993) with a view to reaching agreement on the difference described in that resolution and in Security Council resolution 817 (1993)’.^140^ The legal framework for the settlement of the issue was thus established along with the related UN Resolutions and agreed between the two countries in the Interim Accord. The Interim Accord also secured the future Euro-Atlantic integration of the newly independent state. Greece committed not to block the accession of Macedonia under its provisional name in any regional or international organisation.^141^ The Interim Agreement went even further, stipulating an obligation on Greece to actively support the on-going economic development of Macedonia ‘through international cooperation, as far as possible by a close relationship of [that country] with the European Economic Area and the European Union’.^142^ It further reaffirmed the willingness of the parties to be guided by the principles of international law, including ‘democracy and fundamental freedoms and respect for human rights and dignity, in accordance with the Charter of the United Nations, as well as the Helsinki Final Act, the Charter of Paris for a new Europe and pertinent acts of the Organization for Security and Cooperation in Europe’.^143^ In 2008, however, Greece vetoed Macedonia’s accession to NATO.^144^ The membership of Macedonia to NATO was made explicitly conditional upon the settlement of the name issue contrary to the earlier binding agreement between the two neighbouring states.^145^ That act, as later confirmed by the ICJ, constituted a direct breach of Article 11(1) of the Interim Accord which prohibited Greece from vetoing the accession of Macedonia to international organisations or to institutions of which it itself is a member.^146^

The NATO veto had almost an immediate impact in the EU enlargement context. In fact, the Accession Partnership with Macedonia which preceded the Bucharest Summit had already shifted away from the previous EU approach and listed the solution over the name issue in the short term priorities for the country.^147^ The pressure on the country started increasing. Only two months after the NATO Summit, the Brussels European Council underlined that the ‘maintaining good neighbourly relations, including a negotiated and mutually acceptable solution on the name issue remains essential’^148^ for the further progress of Macedonia towards the EU. Such a reaction was not only inconsistent with the previous EU approach but also ignored the legal significance of the Interim Accord, if not actively encouraging its further violation.

Namely, the breach of the Interim Accord evidently jeopardised the legal framework for settlement of the bilateral dispute between the two countries by shifting the conditions under which the dispute was to be resolved. The abstention of Greece from objections to Macedonia’s prospective EU membership, as envisaged in the Interim Accord, was the only guarantee for preserving equality of the two countries in the process of settling the dispute. And the ICJ judgement confirming Greece’s violation did not change the situation on the ground. Moreover, the Court rejected to order the infringing party to comply with its international obligations, considering that ‘[a]s a general rule, there is no reason to suppose that a State whose act or conduct has been declared wrongful by the Court will repeat that act or conduct in the future, since its good faith must be presumed’.^149^

Contrary to the ICJ’s presumptions, Greece did not comply with its obligations under the Interim Accord. The judgment has also had little impact of note at an EU level despite constant rhetoric that bilateral disputes between countries should be settled in accordance with the principles of international law. It is the international agreement for settlement of the dispute between the neighbouring countries and the ICJ judgement precisely that have been put aside within the EU enlargement process, alongside the fundamental principle of sovereign equality of states in international law.

### Final Settlement of the Dispute and the Rule of Law

Being focused on resolving the bilateral dispute with Greece, rather than implementing any substantial reforms including and particularly with regard to the (lack of) rule of law in the country, Macedonia changed its name on 12 February 2019 when the Treaty of Prespa came into force. The Treaty of Prespa, which terminated the Interim Agreement of 1995, stipulated that: ‘The official name of the Second Party shall be the “Republic of North Macedonia”, which shall be the constitutional name of the Second Party and shall be used erga omnes, as provided for in this Agreement. The short name of the Second Party shall be “North Macedonia”’.^150^ The Treaty of Prespa was signed on 17 June 2018 formally under the UN auspices. That said, it was the inability of Macedonia to move towards NATO and EU that pushed for the final settlement of the dispute. While the Treaty of Prespa marked a new stage in the relationship between the two neighbouring countries, it also meant promotion of politics at the expense of the rule of law under the EU flag.

The signing of the Treaty of Prespa was followed by a referendum in Macedonia, which took place on 30 September 2018, where the Government encouraged citizens to answer ‘yes’ to the referendum question: ‘Are you in favour of membership in the European Union and NATO by supporting the agreement signed on 17 June by the foreign ministers of Greece and Macedonia?’.^151^ The referendum question and a number of other aspects surrounding the referendum provoked an application for assessment of the constitutionality and legality of the Decision to announce a referendum, the nature of the referendum and the referendum question, and required cancellation of the Decision in its entirety on grounds of irremovable formal-legal and substantive-legal defects.^152^ The Constitutional Court decided not to initiate proceedings for assessing the constitutionality and legality of the Decision, rejecting almost all claims of the applicants.^153^ Some of these, however, deserved more serious approach and analysis by the Constitutional Court to ensure the observance of the rule of law.

Thus, one of the arguments of the applicants that was rejected by the Constitutional Court was that the nature of the referendum was not well clarified. In particular, it has been rightly argued that it was not clear from the beginning whether the referendum was of consultative or mandatory nature. The Decision of the Assembly on announcing a referendum was based on Article 73(1) which stipulated that ‘the Assembly decides on issuing notice of a referendum concerning specific matters within its sphere of competence by a majority vote of the total number of Representatives’.^154^ In paragraph 2, that same Article stipulates that ‘[t]he decision of the majority of voters in a referendum is adopted on condition that more than half of the total number of voters voted, and in paragraph 4, it stipulates that ‘[t]he decision made in a referendum is binding’.^155^ Furthermore, a mandatory referendum is announced in cases where a decision on joining or leaving an association or community with other states is made in accordance with Articles 29 of the Referendum Law and Article 120 of the Constitution. The referendum question was certainly formulated in a way to ask voters if they supported membership of the country in the EU, even if a condition for accepting the Treaty of Prespa has been attached to such membership.^156^

With regard to the nature of the referendum and the decision of the referendum, the Constitutional Court opined that it was evident from Article 1 of the Decision of the Assembly on announcing a referendum for ‘consulting’ citizens on the entire territory of the country that the referendum was of consultative nature without discussing extensively the legal basis or the decision of the Government to hold consultative rather than mandatory referendum for a question of significant importance. Having explained the nature of the referendum, the Constitutional Court concluded that the referendum ‘decision does not generate legal, but only a moral obligation for the Assembly to act in accordance with the will of the citizens. It is a constitutional right of the Assembly to decide whether, when and how it will regulate the issue for which the citizens were consulted in such a referendum’.^157^ The explanation of the Constitutional Court came less than two weeks before the referendum day, and could have been hardly heard and understood by the general public who were led to believe by the Prime Minister of the country, Mr. Zoran Zaev, that their will expressed in the referendum will be respected.^158^

Furthermore, it has been argued that the text of the referendum question was ambiguous and vague contrary to Article 15 of the Law on Referendum which stipulates that: (3) If it is voted for more issues, it shall be voted for each issue at a separate ballot; (4) The issue on the ballot must be precisely formulated and unambiguous, so that the citizen at the referendum may answer by “FOR” or “AGAINST”’.^159^ The Constitutional Court rejected this argument too, noting that the referendum question ‘is content of one issue with interconnected, necessary whole, in a historical social context because among its parts there is an internal connection that allows full freedom of expression of citizens’ will as required by the principle of unity of content of the question’.^160^ However, even if not contrary to Article 15 of the Law on Referendum, the referendum question suggesting guaranteed NATO and EU membership if people voted in favour of the Treaty of Prespa has been certainly misleading—an aspect which has been even missed by the dissenting judges.^161^ Indeed, an average citizen who is not aware of the complex enlargement process including the lengthy negotiations procedure and possible future blockades might have reasonably expected that EU accession would have followed immediately after the acceptance of the Treaty of Prespa and that EU accession depended solely on the settlement of the bilateral dispute with Greece. Yet, the Constitutional Court has accepted the referendum question with unbearable easiness noting that ‘consultation with citizens does not mean deciding to join the EU and NATO in the absolute sense of the word’^162^ and also that such consultative referendum ‘in no way infringes any right or obligation for mandatory referendum to be announced in the cases stipulated by the Constitution, and that does not mean taking away the decisive power of the citizens on the issues regulated by the Constitution’.^163^

What the Constitutional Court has missed, however, is the relation between the text of the referendum question and the nature of the referendum. If a referendum question asks voters whether they are in favour of EU membership by accepting certain Treaty, why would an average citizen think that he has not been asked to decide on EU membership ‘in the absolute sense of the word’? Referendum questions do not have asterisks to warn voters that ‘terms and conditions apply’! The referendum question, therefore, violated the fundamental principle of legal certainty of the citizens and intentionally or not, the Constitutional Court failed to prevent violation of the law, putting itself on the side of both the Government and the EU rather than defending the rule of law in the country.

### Disrespect of the referendum results

The referendum unquestionably failed with a turnout as low as 36, 91% (far from the required 50 + 1% by Article 73 of the Constitution), notwithstanding the misleading referendum question. The State Election Commission confirmed that, in accordance with the referendum results, the decision has not been adopted because not more than 50% have casted a vote.^164^ The ‘pro-European’ government, however, interpreted the referendum result as a success arguing that most of the 36, 91% who casted their votes were in favour of the Treaty of Prespa (an overwhelming majority of 94% answered to the referendum question in the affirmative).^165^ However, the picture was not as black and white as presented by the Government. Most of the citizens who were against the Treaty of Prespa boycotted the referendum not to allow for quorum in accordance with the Constitution rather than voting in the negative. Thus, the will of the people was far from what the Government tried to convince everyone in order to justify the ratification of the Agreement. EU equally ignored the referendum results, portraying the process as a success:

‘Following the signature of the historic agreement reached with Greece in June 2018 (also known as the ‘Prespa agreement’), a consultative referendum was organised in September 2018, whereby an overwhelming majority of voters who cast their ballots supported EU and NATO membership by accepting the Prespa agreement’.^166^

Numerous aspects were shifted from their legal framework to provide for ratification of the Treaty: from signing of the Treaty by the Ministers for Foreign Affairs of both parties rather than their Presidents who have the upper hand when it comes to international agreements—an argument which has been also watered-down by the Constitutional Court,^167^ to the refusal of the Macedonian President to sign the Decree for promulgation of the Law on Ratification of the Treaty of Prespa (Law on Ratification) as provided for by the Constitution.^168^

Not less concerning is the fact that the Law on Ratification was sent to the Committee on European Affairs which is dealing with laws for harmonisation of the national legislation with the EU Law (‘Laws with European flag’) rather than to the Committee on Foreign Affairs where it should have been dealt with. This allowed for adoption of the Law on Ratification in a simplified parliamentarian procedure without any serious debate and opposition in the Parliament, even if no EU law was related to the matter. In other words, the simplified procedure for adopting ‘Laws with European flag’ has been misused to avoid much expected complications in the process of ratification in the Assembly. Again, EU remained silent to such violation of the legal procedure which is often practiced in the country when ratification of difficult laws are on the table even if such laws are unrelated or even contrary to EU law.^169^ Finally, the process of passing constitutional amendments in the country has been largely surrounded by allegations of bribery and corruption as well as by painful compromises criticised even by some of the advocates of the entire process.^170^

Last but not least, although helping both parties to reach settlement to their long-lasting dispute, the Treaty of Prespa did not necessarily resolve all issues between the two neighbouring countries.^171^ The interpretation of the Treaty of Prespa might be a major challenge for both parties in the following years.^172^ Even more concerning, however, is the possibility that the Treaty of Prespa might not have solved anything in the long run. As shown in the case of Croatia, bilateral agreements have little value once the candidate country becomes an EU Member State. Should, therefore, North Macedonia eventually access the EU, the Treaty of Prespa may be disputed, if not unilaterally annulled, leaving the EU with no means to tackle that decision.

In fact, the Prespa Treaty has been already problematized by the Macedonian opposition parties who more recently won the local elections in the country and whose councillors rejected to sign their certificates with the new name of the country.^173^ The Macedonian political opposition, which is against the Prespa Treaty, has gained wide support among voters and may well win the next parliamentary elections, putting into question the continuity and enforcement of the bilateral treaty. This is also largely a result of the growing indignation after the country failed to start the accession negotiations once it changed its name owing to new blockades from other EU Member States. After the ratification of the Treaty of Prespa, the beginning of the EU accession negotiations was blocked by France 2019, as well as by The Netherlands and Denmark albeit to a lesser extent, although such blockade was mainly intended for Albania which is in a same ‘enlargement package’ with North Macedonia.^174^ When that blockade was lifted, another bilateral dispute over history, national identity, and language surfaced in 2020, involving Bulgaria and North Macedonia this time and blocking the start of the accession negotiations of the latter.^175^ In other words, the progress of North Macedonia towards the EU has become even more uncertain than before the settlement of the dispute with Greece which promised clearing the path and beginning of accession negotiations with the candidate country. Rather than working towards clarification of the criterion for settlement of bilateral disputes, EU implicitly invited Member States to make use of their membership powers and settle their disputes with neighbouring candidate countries within the enlargement process notwithstanding any derogation of the rule of law.

In the meantime, North Macedonia remains a country with a hybrid democratic regime^176^ and weak rule of law for which the politicisation of the enlargement process largely contributed. The politicised settlement of the name dispute might have not solved the issue between the two countries in the long run. It has rather contributed to further deterioration of the rule of law in the enlargement process and also in the candidate country. The EU disregarded both the Interim Accord and the ICJ judgment, supporting the cloudy referendum process and post-referendum violations of various laws in the country which significantly contributed to further backsliding of the rule of law in the candidate country rather than to any positive reforms. Indeed, even some of the greatest supporters of the referendum have recognised that the price of achieving the change of the name of the country ‘might have been too high in terms of derogation of the rule of law’.^177^ As noted by the conflict analyst and former rule of law officer Eric Manton, ‘[t]here are serious concerns that the way the process was carried out and that painful compromises, made in the name of achieving the result, will be difficult to overcome in order to fully capitalize on the new opportunities of Euro-Atlantic integration’.^178^ In the light of the EU enlargement process, Manton somewhat rhetorically asks: ‘[w]ill some EU member states evaluate the steps taken in this process as counter to EU values and see this as an obstacle for EU aspirations?’.^179^ It is, however, the EU itself that pushed for and applauded the process notwithstanding the means. Evaluating the steps taken in the process would necessarily have to start with an evaluation of the EU’s enlargement condition for settlement of bilateral disputes and EU’s priorities in the Western Balkans. To agree with Manton, EU values including the rule of law have been indeed widely disregarded in the context of the Treaty of Prespa. That said, the Treaty of Prespa would have not seen the light of day without the strong encouragement and pressure from the EU itself.

## A Way Forward

The EU enlargement framework which is largely dominated by politics does not offer an appropriate platform for settlement of bilateral disputes that fall outside the EU law. While by virtue of Article 3(5) TEU and Article 21(1) TEU, the Union is even obliged to make the membership of applicant countries conditional on peaceful settlement of disputes, these should be, however, resolved in full respect of the fundamental principle of sovereign equality of states as between EU Member States.

Indeed, bilateral disputes between EU Member States are not unheard of within the EU. Essentially, there are quite a few unresolved bilateral disputes between Member States—some of which have lasted for decades or even for centuries.^180^ However, Member States have shown little appetite to confront each other, while none of their existing bilateral disputes are regarded today as having the potential of escalating into serious conflicts.^181^ In fact, the Union has been looked upon as contributing to ‘a change in the goals, structure, parties or context of the conflict, which removes or changes the contradiction or incompatibility at its heart’.^182^ Positive relations beyond the unresolved bilateral disputes between Member States result from the changed circumstances, which include the reconceptualization of borders and the prevalence of the common interest over individual national preferences. Should such disputes, however, be brought in connection with EU law, as threatening to undermine the achievements of the integration process, it would be for the EU dispute settlement mechanism to safeguard the compliance of the Member States involved.

That said, the CJEU would not get involved into settlement of bilateral disputes which fall outside the scope of EU law or, as shown in *Slovenia* v *Croatia*, in alleged infringements which are ancillary to settlement of such disputes, under standard infringement procedures. Indeed, being asked what measures could it take to mediate between Italy and France in order to resolve their long standing territorial dispute in the Mont Blank, the European Commission relied on the CJEU ruling in *Slovenia* v *Croatia*, underlining that ‘it is for each Member State to determine the extent and limits of its own territory, in accordance with the rules of public international law. In view of this being a matter of national competence, the Commission does not have the competence to act’.^183^ Settlement of bilateral disputes can be achieved either through mutually agreed arbitration or the ICJ, or by CJEU under Article 273 TFEU which provides for optional jurisdiction of the Court where the dispute is submitted to it under a special agreement between the parties.^184^ Such dispute settlement mechanisms have the potential to provide for fair settlement of bilateral disputes in full respect of sovereign equality of states and should be, therefore, encouraged within the Union and also in the EU enlargement process to bring rule of law on track.

If the bilateral dispute is submitted to the ICJ or to international arbitration, involved states should commit to cooperate in good faith and honour the final decision in the case. Should some of the involved states, however, fail to implement the decision, it is unlikely that the same will be enforced in the EU. The CJEU settlement of bilateral disputes under Article 273 TFEU, therefore, offers better platform for settlement of disputes Member States that fall outside the scope of EU law which are merely connected to the ‘subject matter’ of the Treaties.^185^

Yet, while in the case of arbitration or ICJ, the dispute may be submitted to the chosen body at any stage of the pre-accession process or even after EU accession of the involved candidate country, procedures under Article 273 TFEU require that both involved parties should be EU Member States and, therefore, any settlement of the dispute would be only possible after the EU accession of the involved candidate countries. Making EU accession conditional on future settlement of bilateral disputes under Article 273 TFEU might be, therefore, an optimal solution allowing for a predictable enlargement process.

## Conclusion

Initially intended to transform candidate countries for the better, the rule of law criterion has hardly served its purpose in the EU enlargement to the Western Balkans. In particular, conditionality has been used not so much to achieve stability of institutions guaranteeing democracy and the rule of law, but to achieve political settlement of bilateral disputes contrary to the fundamental principle of sovereign equality of states to which the two elaborated disputes in this article testify.

Indeed, while Croatia achieved EU membership in 2013, the pressing bilateral dispute with Slovenia and mounted pressure from the pre-accession process have had large impact on the later developments and relations between the two neighbouring states, as well as on the backsliding of the rule of law in both countries. The intention of the EU not to get involved into sensitive issues ended up infamously with the CJEU disregarding both the Arbitration Agreement, which the EU previously witnessed and supported in the accession process, and the Final Award. The CJEU’s judgment also confirmed that bilateral treaties that fall outside the EU law will not be interpreted or enforced in the EU even if the conclusion of the treaty was an important accession condition for any of the countries involved. Once both states involved in the dispute become Member States and the EU accession incentive is gone, the enforcement of the bilateral treaty would be largely dependent on the good faith of the involved countries. Actions against Member States brought under Article 258 TFEU or 259 TFEU for alleged infringements of EU law that are ancillary to settlement of bilateral disputes would be dismissed by the CJEU due to lack of jurisdiction. Put differently, the case of Croatia shows that the pre-accession condition for settlement of bilateral disputes which often halts the enlargement process contrary to the rule of law principle, losses both its significance and its aim post-accession, notwithstanding the outcome of the dispute settlement process. More importantly, however, the somewhat forced agreement for the settlement of the bilateral dispute has not contributed to the strengthening of the rule of law in the pre-accession process. Quite to the contrary, it has made the enlargement process less predictable by putting ambitions and domestic policies of Member States before the established Copenhagen criteria and the international principle of sovereign equality of states.

The situation in North Macedonia is much worse. More than 16 years after receiving candidate status, the rule of law in the country remains weak despite constant recommendations of the European Commission for opening of the accession negotiations since 2009. Rather than enforcing Copenhagen criteria and making the pre-accession progress of the country conditional on the strengthening of the rule of law, the EU has invested enormous efforts in what was essentially a political bilateral dispute between states, ignoring if not supporting the violation of the rule of law in the candidate country. The EU has first ignored the Interim Accord between Greece and Macedonia, then it ignored the ICJ judgement confirming breach of the Interim Accord by one of the sides involved, and finally it supported and cherished the change of the name of the candidate country despite the many irregularities of the process resulting in further deterioration of the rule of law. Yet, even the high price of sacrificing the law has not brought joy to the candidate country but opened the way to more blockades and bilateral disputes. This has added further to the lack of predictability of the enlargement process, putting future reforms in the field of rule of law in the country into question. Moreover, the EU cannot secure the continuity and enforcement of the Prespa Agreement or of any other bilateral treaty that falls outside the scope of EU law once and if North Macedonia becomes an EU Member State. The *pacta sunt servanda* principle does not apply to bilateral treaties between Member States owing to EU law supremacy over such treaties.

The current enlargement framework which includes the rather subjective, imprecise, vague and unverifiable condition for settlement of bilateral disputes, does not only undermine the rule of law as a fundamental part of the enlargement regulation and causes further deterioration of the rule of law in the involved candidate countries, but is also ineffective. Permanent solution to bilateral disputes that fall outside the EU law cannot be achieved through political pressure and power exercise in the pre-accession process but require strong leadership and respect for the rule of law notwithstanding the framework. While the settlement of only two bilateral disputes has been discussed in this article, remaining unresolved disputes between other Western Balkan countries and candidate countries may prove equally problematic. Should the EU continue to apply political conditionality for settlement of such disputes in the pre-accession process, i.e. within a framework of asymmetric relations, there will be hardly any progress of the rule of law in the candidate countries.

A genuine reconsideration of the requirement for settlement of bilateral disputes blocking the enlargement process is urgently needed to bring rule law on track. While international courts and arbitrations may offer a good platform for settlement of disputes where both sides freely agree on the mechanism used, enforcement of decisions of international judicial and non-judicial bodies may be challenging. Settlement of bilateral disputes by the CJEU under Article 273 TFEU, once all involved parties have become Member States, might be, therefore, an optimal solution. In such case, the EU would be able to frame settlement of bilateral disputes as an ‘objective, precise, detailed, strict and verifiable’ enlargement condition with credible post-accession consequences. Infiltrating Article 273 TFEU in the enlargement framework would correct the level of unbalanced powers between Member States and candidate countries, and would presumably contribute to decrease in the number of bilateral disputes.

Until then, the EU commitment towards the Western Balkan region and strengthening of the rule of law in the pre-accession process remains only an empty rhetorical commitment.

## Footnotes

1 Timmermans (2015); cf Magen and Pech (2018).

2 European Commission ‘2021 Rule of Law Report: The rule of law situation in the European Union’, COM (2021) 700 final (EC 2021 Rule of Law Report), 1. Cf Pech, Grogan et al. (2020).

3 EC 2021 Rule of Law Report.

4 Blauberger and van Hüllen (2021).

5 Janse (2018).

6 The incentives for other outsiders, e.g. for countries that participate in the European Neighbourhood Policy and beyond, are smaller and less attractive, ranging from financial support to cooperation agreements, to relaxation of visa requirements. *See* e.g. Blauberger and van Hüllen (2021).

7 Basheska (2014); Basheska (2015a); Basheska and Kochenov (2015c).

8 Linderfalk (2020); ILC Report, A/74/10, 2019, chap. V, Conclusion 23 (13); cf. Tladi (2021).

9 Article 2(1), Charter of the United Nations (adopted 26 June 1945, in force 24 October 1945) 59 Stat. 1031 (UN Charter).

10 *See* e.g. the most recent Special Report of the European Court of Auditors: ‘EU support for the rule of law in the Western Balkans: despite efforts, fundamental problems persist’ (10 January 2022) < https://www.eca.europa.eu/Lists/ECADocuments/SR22_01/SR_ROL-Balkans_EN.pdf?fbclid=IwAR3R4FYCLPiDe5xDeyo_c4vODxj5m3paLk372bs9FiuNghd2kvSamN6y9jI > accessed 10 January 2022 (Special Report of the European Court of Auditors).

11 Special Report of the European Court of Auditors.

12 European Commission, ‘A credible enlargement perspective for and enhanced EU engagement with the Western. Balkans’ COM (2018) 65 final (EC 2018 Communication), p. 3.

13 EC 2018 Communication, p. 3.

14 Special Report of the European Court of Auditors, p. 4.

15 Special Report of the European Court of Auditors, p. 4.

16 European Commission, ‘Enhancing the accession process—A credible EU perspective for the Western Balkans’ COM (2020) 57 final (EC 2020 Communication); European Commission, ‘2021 Communication on EU Enlargement Policy’ COM (2021) 644 final (EC 2021 Communication).

17 At the time of writing, Albania and North Macedonia have not started their accession negotiations with the EU due to a blockade from Bulgaria over an unresolved bilateral dispute with North Macedonia, although the Macedonian special envoy for resolving the dispute between the Skopje and Sofia authorities, Vlado Buchkovski, most recently informed that the veto will be lifted in the following months, while the two new prime ministers of the countries agreed to improve relations, *see* Nikolov (2022) and Testorides (2022) respectively. Being linked together in the enlargement process, Albania and North Macedonia share the same destiny even if no bilateral disputes prevent Albania currently from starting its negotiations with the EU.

18 ‘Republic of Macedonia’ changed its name to ‘Republic of North Macedonia’ on 12 February 2019. The change of the name of the country has been discussed in more detail in Sect. 5 of this article. The new name of the country is used in this article for discussion of the circumstances after the country officially changed its name.

19 These include but are not limited to: Croatia-Serbia dispute on their border in the Danube area, for which the (now) President of Croatia noted back in 2014, while he was still a Prime Minister of that country, that settlement should be made condition for Serbia during the accession negotiations, *see* Bošnjak and Ciglenečki (2014), *see* more recent discussion about the possibility for blockade over the border dispute Žeželj (2021); Moreover Croatia and Serbia cannot agree on several areas that none of the two countries claims, two of which have been even proclaimed Free Republics (Liberland and Verdis), *see* Jenkins (2016), Kruljac (2021) and the official website of Free Republic of Verdis < https://www.verdisgov.org/ > ; Bosnia-Croatia disputed sections of the boundary, and primarily the one related to maritime access, *see* Brezar (2021); a Croatia-Montenegro sea border dispute at the Prevlaka peninsula Kajosevic (2020), to mention but a few bilateral disputes that may be problematized in the EU enlargement process.

20 General Affairs and External Relations Council (Luxembourg, 16 June 2002), ‘Thessaloniki Agenda for the Western Balkans – Moving towards European Integration annexed to the Thessaloniki European Council’s Presidency Conclusions (Thessaloniki Agenda)’, 10,369/03 (Presse 166).

21 Emmott (2021).

22 Siebold, Sekularac and Emmott (2021).

23 Basheska and Kochenov (2015d), p. 392.

24 EC 2020 Communication, p. 5 (emphasis in original).

25 EC 2020 Communication, p. 5 (emphasis in original).

26 EC 2020 Communication, p. 5.

27 Uilenreef (2010), p. 28.

28 Kochenov (2008), p. 312.

29 Basheska (2014).

30 European Commission ‘Agenda 2000: for a stronger and wider Union’ COM (1997) 2000 final (Agenda 2000), p. 51.

31 Opinion on Greek application for membership, EC Bull Supp 2/76, para. 6 (Opinion on Greek application).

32 The European Parliament has been more straightforward as to the nature of the requirement, if not to its substance, stating that ‘any acceding state should resolve its main bilateral problems and major disputes with neighbours, (…) before it can join the Union’, European Parliament Resolution on Enlargement: Policies, Criteria and the EU’s Strategic Interests 2012/2025 (INI), 22 November 2012 (EP Resolution on Enlargement 2012).

33 European Commission, ‘Enlargement Strategy and Main Challenges 2011–2012’ (Communication) COM (2011) 666 final (EC 2011 Communication).

34 EC 2011 Communication.

35 EC 2021 Communication, p. 25, para. 1 (emphasis in original).

36 EC 2020 Communication, p. 1.

37 EC 2020 Communication, p. 2.

38 EC 2020 Communication, p. 1 (emphasis in original).

39 EC 2020 Communication, p. 1 (emphasis in original).

40 EC 2020 Communication, p. 1 (emphasis in original).

41 EC 2020 Communication, p. 1 (emphasis in original).

42 EC 2020 Communication, p. 1 (emphasis in original).

43 EC 2020 Communication, p. 2 (emphasis in original).

44 EC 2020 Communication, p. 2 (emphasis in original).

45 EC 2020 Communication, p. 2 (emphasis in original).

46 EC 2020 Communication, p. 2 (emphasis in original).

47 EC 2020 Communication, pp. 2‒3 (emphasis in original).

48 EC 2020 Communication, p. 3 (emphasis in original).

49 EC 2020 Communication, p. 3 (emphasis in original).

50 EC 2020 Communication, p. 4 (emphasis in original).

51 EC 2020 Communication, p. 4 (emphasis in original).

52 EC 2020 Communication, p. 4 (emphasis in original).

53 EC 2020 Communication, p. 4 (emphasis in original).

54 *See* Sect. 4 of this article.

55 European Commission, ‘Commission welcomes the green light to opening of accession talks with Albania and North Macedonia’ press (25 March 2020), available at: < https://ec.europa.eu/commission/presscorner/detail/pt/ip_20_519 > accessed 7 January 2022 (European Commission press).

56 European Commission press.

57 European Commission press.

58 Instead, the EU candidate countries from the Western Balkans created their own ‘union’ in response to the stalled EU enlargement process, which has been, however, already criticised by EU diplomats, e.g. *Exit* (2021).

59 EC 2021 Communication.

60 EC 2021 Communication, p. 22.

61 EC 2021 Communication, p, 25, para. 3.

62 EC 2021 Communication, p. 22.

63 EC 2021 Communication, p. 23.

64 The dissolution of Yugoslavia brought to surface several disputes between Croatia and Slovenia, some of which were used as a leverage by Slovenia in the EU enlargement process. One such dispute involved disagreement between the two neighbouring states over money owed by a Slovenian bank to Croatian depositors. In 2010, Slovenia blocked the closure of the free movement of capital chapter of Croatia and later on conditioned the ratification of Croatia’s Accession Treaty with a solution of the dispute in accordance with international law. The obstacle has been only removed with the signing of a memorandum of understanding by both countries under which the involved parties agreed to take the case to the Bank for International Settlements in accordance with the Agreement on Succession. *See* in more detail Mozina (2014).

65 Solomou (2017).

66 For an overview of the border dispute, *see* Uilenreef 2010, pp. 15‒22.

67 Principle 13 of the Negotiating Framework for Croatia, Luxembourg, 3 October 2005, < http://ec.europa.eu/ enlargement/pdf/croatia/st20004_05_hr_framedoc_en.pdf > last accessed 11 May 2014 (Negotiating Framework for Croatia).

68 Principle 13 of the Negotiating Framework for Croatia.

69 Avbelj and Letnar Černič (2007), p. 2.

70 Uilenreef (2010), p.17.

71 Hillion (2011), p. 201.

72 Arbitration Agreement between the Government of the Republic of Slovenia and the Government of the Republic of Croatia (signed 4 November 2009, in force 29 November 2010) (Arbitration Agreement).

73 Article 9(1), Arbitration Agreement.

74 Article 9(2), Arbitration Agreement.

75 Arbitration Agreement between the Government of the Republic of Slovenia and the Government of the Republic of Croatia, signed 4 November 2009, < https://www.assidmer.net/doc/Arbitration_Agreement_Croatia_Slovenia.pdf > (Arbitration Agreement) accessed 7 January 2022.

76 Treaty of Accession of Croatia [2012] OJ L112/10.

77 European Commission, Launch of the arbitration process between Slovenia and Croatia, IP/12/25, Press Release, 17 January 2012.

78 Article 7(2), Arbitration Agreement.

79 Articles 6 and 11, the Permanent Court of Arbitration Optional Rules for Arbitrating Disputes between Two States.

80 Article 10(1), Arbitration Agreement.

81 Article 10(2), Arbitration Agreement.

82 Statement available in: ‘Arbitration between the Republic of Croatia and the Republic of Slovenia’. < http://www.mvep.hr/files/file/dokumenti/arbitraza/en/150820-letter-from-fm-pusic-to-mr-pulkowski-pca-30042015.pdf > last accessed 27 November 2021 (Statement).

83 Statement.

84 Statement. *See* also the text of the position of the Slovenian Assembly from 4 February 2013 in Slovenian: < http://www.mvep.hr/files/file/dokumenti/arbitraza/en/150820-sklep-drzavneg-zbora-04022013.pdf > last accessed 27 November 2021. For unofficial translation in English, *see* < http://www.mvep.hr/files/file/dokumenti/arbitraza/en/150820-conclusion-of-the-national-assembly-of-slovenia-04022013.pdf > last accessed 27 November 2021.

85 The letter of the Tribunal is available at: < http://www.pcacases.com/web/sendAttach/1307 > last accessed 27 November 2021 (Tribunal letter).

86 Tribunal letter.

87 The audio recordings are available at: < http://www.newsweek.rs/region/53276-njuzvik-ekskluzivno-otkriva-poslusajte-audio-zapis-kako-slovneci-preotimaju-hrvatima-piranski-zaliv-video.html > and at: < http://www.vecernji.hr/hrvatska/ekskluzivno-donosimo-razgovor-arbitra-i-slovenske-strane-poslusajte-snimke-1015908 > . Transcripts of recordings in English are available at: < http://www.mvep.hr/files/file/dokumenti/arbitraza/en/150820-excerpts-from-recordings-between-dr-sekolec-and-mr-drenik-14082015.pdf > all last accessed 8 September 2021.

88 Statement available at the official website of the Ministry of Foreign and European Affairs at: < http://www.mvep.hr/en/other/termination-of-the-arbitration-process/ > last accessed 8 September 2021.

89 *See* Press Release of the Tribunal < http://www.pcacases.com/web/sendAttach/1310 > last accessed 8 September 2021.

90 Letter available at: < http://www.mvep.hr/files/file/dokumenti/arbitraza/en/150820-letter-from-fm-pusic-to-mr-pulkowski-pca-24072015.pdf > last accessed 8 September 2021 (Lett.

91 Ruling available at: < http://www.mvep.hr/files/file/dokumenti/arbitraza/en/150820-ruling-of-the-croatian-parliament-29072015.pdf > last accessed 8 September 2021.

92 Available at: < http://www.mvep.hr/files/file/dokumenti/arbitraza/en/150820-decision-of-the-croatian-government-30072015.pdf > last accessed 8 September 2021.

93 Decision available at: < http://www.mvep.hr/files/file/dokumenti/arbitraza/en/151002-letter-from-pm-milanovic-to-president-juncker-01102015.pdf > and at: < http://www.mvep.hr/en/info-servis/press-releases/fm-kovac-croatia-will-not-attend-arbitration-hearing,25727.html > last accessed 8 September 2021 (Decision for termination of the Agreement).

94 Decision for termination of the Agreement.

95 Decision for termination of the Agreement.

96 Decision for termination of the Agreement.

97 Para. 220, Partial Award (30 June 2016), available at: < https://pcacases.com/web/sendAttach/1787 > accessed 7 January 2022 (Partial Award).

98 Para. 175, Partial Award.

99 Para 225, Partial Award. Cf Ilic 2017 and Runjić (2019).

100 Para 225, Partial Award.

101 *See* Press release on Arbitral Tribunal’s decision of 30 June 2016 < http://www.mvep.hr/en/info-servis/press-releases/press-release-on-arbitral-tribunal%E2%80%99s-decision-,25852.html > last accessed 27 November 2021 (Press release on Arbitral Tribunal’s decision).

102 Press release on Arbitral Tribunal’s decision.

103 PCA Case no. 2012–04, Final Award (29 June 2017), available at: < https://pcacases.com/web/sendAttach/2172 > last accessed 27 November 2021, A summary of the Final Award is available at: < https://pcacases.com/web/sendAttach/2175 > accessed 7 January 2022.

104 *See* Statement of the Government of the Republic of Croatia on the Arbitral Tribunal’s award of 29 June 2017 < https://vlada.gov.hr/statement-of-the-government-of-the-republic-of-croatia-on-the-arbitral-tribunal-s-award-of-29-june-2017-23774/23774 > accessed 7 January 2022.

105 The entire dispute has been extensively discussed by Bickl 2021.

106 *See* < https://www.neweurope.eu/article/croatias-bid-oecd-membership-blocked-slovenia-hungary/ > accessed 7 January 2022.

107 Case T-626/17 *Republic of Slovenia* v *European Commission*, ECLI:EU:T:2020:402.

108 Bickl (2021), p. 229.

109 *See* Opinion of Advocate General Pikamäe of 11 December 2019, para. 87: ‘The Commission, relying on the order of 23 October 2002 in *Austria v Council*, contends that to produce internal documents of that nature in proceedings before the Court, unless such production has been authorised by the institution concerned or ordered by the Court, would be contrary to the public interest in institutions being able to receive the advice of their legal services, given in full independence. According to the Commission, the legal opinion at issue is an internal document that was not intended to be published and to which the public was not given access. The Commission states that production of the opinion in proceedings before the Court was not authorised. The legal opinion at issue should therefore be removed from the case file’ (footnotes omitted). See also paras 64–73 of C-457/18, *Republic of Slovenia* v *Republic of Croatia*, ECLI:EU:C:2020:65 (*Slovenia* v *Croatia*).

110 Müller (2018).

111 Müller (2018).

112 Question for written answer E-000513/2020 to the Commission, Rule 138 (29 January 2020), < https://www.europarl.europa.eu/doceo/document/E-9-2020-000513_EN.html > accessed 7 January 2022 (Question for written answer).

113 Question for written answer.

114 *Slovenia* v *Croatia*, para. 1.

115 *Slovenia* v *Croatia*, para. 1.

116 *Slovenia* v *Croatia*, para 75.

117 *Slovenia* v *Croatia*, para 75.

118 *Slovenia* v *Croatia*, para 76.

119 *Slovenia* v *Croatia*, para 77.

120 *Slovenia* v *Croatia*, para 104.

121 *Slovenia* v *Croatia*, para 104.

122 McGarry (2021). *See Slovenia* v *Croatia*, para. 92.

123 *Slovenia* v *Croatia*, para. 102.

124 *Slovenia* v *Croatia*, para. 104.

125 *Slovenia* v *Croatia*, para. 103. *See* also McGarry (2021).

126 *See* eg Martin (1990); Tanzi (1995).

127 Case C-132/09, *European Commission* v *Kingdom of Belgium*, ECLI:EU:C:2010:562, para. 44.

128 *Slovenia* v *Croatia*, para. 109.

129 Partial Award, para. 225.

130 Partial Award, para. 225.

131 McGarry (2021).

132 McGarry (2021); Basheska (2014).

133 *Slovenia* v *Croatia*, para. 107.

134 *See* in particular European Commission observations in PCA Case No. 2008–13 (*Eureko BV* v. *the Slovak Republic*) and relevant CJEU cases: Case 10/61 *Commission* v. *Italy*, ECLI:EU:C:1962:2; Case C-235/87 *Matteucci* v. *Communauté française of Belgium et. al*, ECLI:EU:C:1988:460 (para. 22); Case 3/91, *Exportur SA* v. *Lor SA and Confiserie du Tech SA*, ECLI:EU:C:1992:420 (para. 8).

135 E.g. Gašperin (2020); Kassoti (2020).

136 Cf Kochenov (2021).

137 For a brief summary of the arguments of the two states and a chronological record of the developments regarding the dispute *see* CoE: Parliamentary Assembly *–* Working papers *–* 2008 Ordinary Session (Second part) 14–18 April *–* Volume III (2008) 37–41.

138 Basheska (2015b)

139 Basheska (2014); Basheska (2015b).

140 UN Interim Accord Between the Hellenic Republic and the FYROM UN Doc 95 − 27,866 of 13 September 1995 (Interim Accord), Article 5(1).

141 Interim Accord, Article 11(1).

142 Interim Accord, Article 11(2).

143 Interim Accord, Preamble.

144 NATO Summit (2–4 April 2008).

145 Point 20 of the Bucharest Summit Declaration, available at: < https://www.nato.int/cps/en/natolive/official_texts_8443.htm > accessed 7 January 2022.

146 This violation by Greece was later confirmed in a judgement of the ICJ of 9 December 2011. *See* Application of the Interim Accord of 13 September 1995 (*The former Yugoslav Republic of Macedonia* v. *Greece*) [2011] ICJ Rep 2011 (*Macedonia* v *Greece*).

147 Council Decision, 2008/212 on the principles, priorities and the conditions contained in the European Partnership with the Former Yugoslav Republic of Macedonia and repealing Decision 2006/57/EC [2008] OJ L80/32 (Council Decision 2008/212).

148 Brussels European Council (19, 20 June 2008) Presidency Conclusions, para. 56.

149 *Macedonia* v. *Greece*, para. 168.

150 Article 1(3)(a), ‘Final Agreement for the settlement of the differences as described in the United Nations Security Council Resolutions 817 (1993) and 845 (1993), the termination of the Interim Accord of 1995, and the establishment of a strategic partnership between the Parties’.

151 Decision to announce a referendum no.08–4666/1, adopted by the Assembly of the Republic of Macedonia on 30 July 2018, ‘Official Gazette of the Republic of Macedonia’ no.140/2018 (Decision to announce a referendum).

152 Resolution U. no.88/2018, 90/2018, 91/2018 (19 September 2018).

153 Separate opinions on the Resolution U.no.88/2018, 90/2018, 91/2018. Cf Judge Gosheva and Judge Josifovski.

154 Decision to announce a referendum; Constitution of the Republic of North Macedonia < https://vlada.mk/sites/default/files/dokumenti/zakoni/the_constitution_of_the_republic_of_north_macedonia_containing_the_valid_constitutional_provisions_in_force_as_amended_by_constitutional_amendments_i-xxxvi.pdf > accessed 7 January 2022.

155 Constitution of the Republic of North Macedonia.

156 Cf Judge Gosheva and Judge Josifovski.

157 Resolution U.no.88/2018, 90/2018, 91/2018 (19 September 2018), para. 6.

158 *See* e.g. an interview of the Prime Minister given to a Greek TV before the referendum: < https://www.youtube.com/watch?v=3pEuRSWFEQA&t=162s > accessed 7 January 2022.

159 Law on Referendum and Other Forms of Direct Vote of Citizens (‘Official Gazette of the Republic of Macedonia’ no. 81/2005).

160 Resolution U.no.88/2018, 90/2018, 91/2018 (19 September 2018), para. 6 (Resolution U.no.88/2018, 90/2018, 91/2018).

161 Cf Judge Gosheva and Judge Josifovski.

162 Resolution U.no.88/2018, 90/2018, 91/2018, para. 6.

163 Resolution U.no.88/2018, 90/2018, 91/2018, para. 6.

164 The ‘Report on the final results of the voting at the referendum on a state level’ of 20 September 2018 is available at: < https://dejure.mk/zakon/izveshtaj-za-konechnite-rezultati-od-glasanjeto-na-referendumot-na-drzhavno-nivo-30-septemvri-2018-godina > accessed 7 January 2022 (Report on the final results of the voting at the referendum).

165 Report on the final results of the voting at the referendum.

166 European Commission, ‘Key findings of the 2019 Report on North Macedonia’ (29 May 2019) available at: < https://ec.europa.eu/commission/presscorner/detail/en/COUNTRY_19_2777 > accessed 7 January 2022.

167 Resolution U.no.88/2018, 90/2018, 91/2018, para. 5.

168 Karakamisheva and Saveski (2018), pp. 36‒38.

169 Tumanovska (2019); Tanevski (2021).

170 Rose (2019).

171 E.g. in the more recent European Football Championship the use of the name ‘Macedonia’ by the Football Federation was problematised testifies. See also Chryssogelos and Stavrevska (2019).

172 Holroyd (2021); Claus (2021).

173 *See* e.g. 360° with regard to < https://360stepeni.mk/izleguvane-od-nato-ponishtuvanje-na-priznavanjeto-na-kosovo-za-shto-se-zalaga-levitsa-eden-od-partnerite-vo-mozhnoto-novo-mnozinstvo/ > ; see also NovaTV. < https://novatv.mk/gordana-silanovska-ke-bara-ponishtuvane-na-prespanskiot-dogovor/ > accessed 7 January 2022.

174 Tidey, Chadwick and Koutsokosta (2019).

175 Tsolova (2020).

176 See the most recent Freedom House Report available at: < https://freedomhouse.org/country/north-macedonia/nations-transit/2020 > accessed 7 January 2022.

177 Rose (2019).

178 Rose (2019).

179 Rose (2019).

180 These include but are not limited to: the Mont Blanc summit dispute between France and Italy; Rockall, uninhabited island in the North Atlantic Ocean disputed by Denmark, Iceland, Ireland, and the UK, *see* Derrig (2021); dispute between Germany and the Netherlands with regard to the exact course of border through Dollart bay, *see* Gerard (1987); dispute between Austria, Germany and Switzerland over the boundaries between the three states in Lake Constance, *see* Frowein (1990); dispute between Portugal and Spain regarding the status of Olivenza (Black 1979), to mention only some of the unsettled bilateral disputes between EU Member States.

181 Anderson (2000).

182 Miall (2007), p. 14, distinguishes between four different types of change that could lead to transformation of conflicts: actor transformation (change in the existing parties or appearance of new ones); issue transformation (change in the priority of interests and more particularly ‘[reducing] the relative importance of issues on which antagonism exists and [emphasizing] the issues on which commonality prevails’); rule transformation (change in the norms in interstate relations); and structural transformation (change in the relationship between actors). Two additional types of changes have been added by other scholars: Ramsbotham, Voodhouse and Miall (2011), p. 175, consider changes in the social, regional or international context which surround a conflict as important. Diez et al. (2008), p. 7, add changes to the communication that constructs the conflict. McCall (1998), p. 392, argues that ‘[b]y challenging the power of the nation-state centre, initiating supranational citizenship and encouraging a multilevel system of government, the EU polity is beginning to display a reconstructivist impulse’.

183 Question reference: E-005844/2020 (footnotes omitted);

184 Basheska (2021).

185 *See* Basheska (2021); Nowak (2020); Peers (2012); Cremona (2012); Schermers and Waelbroeck (2001), p. 643.
